# Drug-induced polycystic ovary syndrome: a real-world pharmacovigilance study based on the FAERS database

**DOI:** 10.3389/fendo.2025.1671511

**Published:** 2025-11-24

**Authors:** Huiping Zhang, Man Di, Yu Wang, Yingying Ma, Yulu Gou, Zhuo Zhou

**Affiliations:** 1 Department of Obstetrics and Gynecology, Northwest University First Hospital, Xi’an, Shaanxi, China; 2 Department of Obstetrics and Gynecology, Tangdu Hospital, Air Force Medical University, Xi’an, China; 3 The Department of Obstetrics and Gynecology, Affiliated Hospital of Yan’an University, Yan’an, Shaanxi, China

**Keywords:** polycystic, ovary, syndrome, FAERS, adverse, events, disproportionality analysis, pharmacovigilance

## Abstract

**Objective:**

Previous studies have shown an association between polycystic ovary syndrome (PCOS) and the use of various medications. However, there is still a lack of systematic research exploring this relationship in depth. This study aims to identify and evaluate drugs that may influence the risk of PCOS using the US FDA Adverse Event Reporting System (FAERS) database.

**Methods:**

Adverse events (AEs) related to drug-induced PCOS were retrieved from the FAERS database (Q1–2014 to Q4 2024). Four statistical methods (ROR, PRR, BCPNN, and MGPS) were used for imbalance analysis to identify drugs significantly associated with PCOS risk. Additionally, a latency (TTO) analysis was conducted to assess the timing of onset and the risk characteristics of PCOS-related adverse reactions.

**Results:**

This study identified 18 drugs significantly associated with PCOS-related AEs from a total of 1,516 cases through imbalance analysis. These drugs span various categories, including respiratory, antipsychotic, and anticonvulsant medications. Among them, Mecasermin (ROR = 67.54) and Ciclesonide (ROR = 62.10) presented the highest risk, followed by Valproic acid (ROR = 20.78) and Olanzapine (ROR = 10.27). Adverse events were most commonly observed either after 360 days of medication use or within 30 days. The median time to onset for the top three drugs with the highest signal frequency was as follows: Olanzapine (155.5 days), Quetiapine (335 days), and Valproic acid (905 days).

**Conclusion:**

This study is the first large-scale, systematic exploration of drug signals related to PCOS using the FAERS database. The drugs identified are primarily associated with the nervous system, followed by respiratory system medications and other types of drugs. These findings provide new warning evidence and references for clinical drug safety, suggesting that enhanced monitoring of female patients should be implemented when prescribing such drugs.

## 1 Introduction

PCOS is a common endocrine disorder of the female reproductive system, with an estimated prevalence ranging from 4% to 12% (1). Its typical clinical manifestations include irregular menstruation, hyperandrogenism, and polycystic ovarian changes (2). Additionally, PCOS increases the risk of various long-term complications in women, including infertility, type 2 diabetes, atherosclerosis, dyslipidemia, and cardiovascular diseases (3–6). The exact pathogenesis of PCOS is not yet fully understood, but it is widely believed that genetic, environmental, and lifestyle factors play a major role in its development (7). It is worth noting that drug side effects can be a potential risk factor for multiple diseases and may also extend to the realm of PCOS (8). Existing research indicates that certain hormonal drugs, antipsychotics, antiepileptic drugs, and others may interfere with ovarian function in women through complex physiological mechanisms, thereby triggering symptoms related to PCOS (9–11).

Current studies on drug-related PCOS mainly come from clinical trials and observational research, but these studies have limited sample sizes, disease coverage, and drug evaluation ranges. In real-world settings, large-scale, comprehensive research and sufficient data on drug-induced PCOS are still lacking. The FAERS database, one of the largest drug adverse event databases in the world, can identify potential associations between drugs and PCOS in a broad patient population. Research based on FAERS effectively overcomes the limitations of traditional clinical trials, such as small sample sizes and short observation periods (12). To date, there has been no extensive analysis from FAERS on drugs associated with an increased risk of PCOS. Therefore, this study aims to analyze FAERS data to identify potential risk signals of drugs that may increase the risk of PCOS, assess the associated risk levels, and systematically examine the relationship between these drugs and PCOS. This analysis will help clinicians assess the risk of PCOS when choosing treatment options and provide guidance for future basic research on potential mechanisms, ultimately advancing drug risk assessment and management strategies.

## 2 Materials and methods

### 2.1 Data source and preprocessing

The data for this study was sourced from the FAERS database, which can be downloaded from the FDA website (https://fis.fda.gov/extensions/FPD-QDE-FAERS/FPD-QDE-FAERS.html). The database offers two download formats: ASCII and XML. For statistical analysis, this study downloaded the raw ASCII data from the first quarter of 2014 to the fourth quarter of 2024. The dataset includes patient demographics, drug information, adverse events, outcomes, report sources, treatment duration, and usage indications. A deduplication protocol was implemented according to FDA guidelines (13). Key fields such as PRIMARYID, CASEID, and FDADT were meticulously extracted and organized from the source database. For reports with the same CASEID, the entry with the latest FDADT was retained. For records with identical CASEID and FDADT, the record with the highest PRIMARYID was selected. In cases where both CASEID and FDADT were identical, the record with the highest PRIMARYID was selected (as shown in **Table 1**).

**Table 1:** Example of duplicate report removal criteria.

CASEID	FDA_DT	PRIMARYID	Delete
4070800	20040113	4271953	Yes
4070800	20040113	4271960	Yes
4070800	20040130	4283861	Yes
4070800	20040308	4314767	No

### 2.2 Adverse reaction identification

In this study, MedDRA dictionary (MedDRA 27.1) preferred terms (PTs) were used to encode adverse event names in the FAERS database. Standardized queries were used to identify PTs related to Drug-induced Polycystic Ovary Syndrome, and the term “Drug-induced Polycystic Ovary Syndrome” was selected for analysis. Cases related to this PT were extracted from the DEMO file using their PRIMARYID, and duplicate reports were removed using a deduplication algorithm based on patient ID and report event time, ensuring that each event was counted only once. For the drug involvement analysis, only cases labeled as “primary suspected drug” were included, while entries labeled as “secondary suspected drug,” “concomitant drug,” or “interaction drug” were excluded to reduce result uncertainty.

### 2.3 Statistical analysis

This study employed four methods to identify potential drug-event associations: Reporting Odds Ratio (ROR) (14), Proportional Reporting Ratio (PRR) (15), Bayesian Confidence Propagation Neural Network (BCPNN) (16), and Multi-item Gamma Poisson Shrinker (MGPS) (17). These methods evaluate the statistical relationship between a specific drug and a specific adverse event (AE) by calculating the relative frequency of target drug-related target AEs in the database over a period of time. a refers to the number of reports containing both the target drug and target AE. b refers to the number of reports containing the target drug and other drug AEs. c refers to the number of reports containing other drugs and the target AE. d refers to the number of reports containing other drugs and other drug AEs (as shown in **Table 2**). The formulas for these four methods are presented in **Table 3**. Drugs that met the criteria from all four methods were included in the study, indicating a significant association with Drug-induced Polycystic Ovary Syndrome.

**Table 2:** Contingency table for proportional imbalance measurement method.

Types of drugs	Target adverse event reports	Other adverse event reports	Total
Target drug	a	b	a+b
Other drugs	c	d	c+d
Total	a+c	b+d	a+b+c+d

**Table 3:** Four main algorithms for evaluating the potential association between drugs and drug-induced polycystic ovary syndrome.

Algorithms	Equation	Criteria
ROR	$ROR=\frac{ad}{bc}$ $95\%CI=e^{\ln(ROR)\pm \sqrt[{1.96}]{\left(\frac{1}{a}+\frac{1}{b}+\frac{1}{c}+\frac{1}{d}\right)}}$	95% CI (lower limit) > 1, a ≥ 3
PRR	$PRR=\frac{a/(a+b)}{c/(c+d)}$ $95\%CI=e^{\ln(PRR)\pm \sqrt[{1.96}]{\left(\frac{1}{a}-\frac{1}{a+b}+\frac{1}{c}-\frac{1}{c+d}\right)}}$ χ2=[(ad−bc)^2](a+b+c+d)/[(a+b)(c+d)(a+c)(b+d)]	a ≥ 3, 95% CI (lower limit) > 1, and PRR ≥ 2
BCPNN	$IC=\log_{2}\frac{a(a+b+c+d)}{\left(a+c)(a+b\right)}$	IC-2SD >0
MGPS	$\begin{array}{l} EBGM=a(a+b+c+d)/(a+c)/(a+b)\\ 95\%CI=e^{\ln(EBGM)\pm \sqrt[{1.96}]{\left(\frac{1}{a}+\frac{1}{b}+\frac{1}{c}+\frac{1}{d}\right)}} \end{array}$	EBGM05>2

ROR, Reporting Odds Ratio; PRR, Proportional Reporting Ratio; BCPNN, Bayesian Confidence Propagation Neural Network; MGPS, Multi-item Gamma Poisson Shrinker; EBGM, Empirical Bayesian Geometric Mean; CI, Confidence Interval; χ2, Chi-square; IC, Information Component; IC025, the lower limit of the 95% one-sided confidence interval for IC; EBGM05, the lower limit of the 95% CI for EBGM.

## 3 Results

### 3.1 Data retrieval results

**Figure 1:**
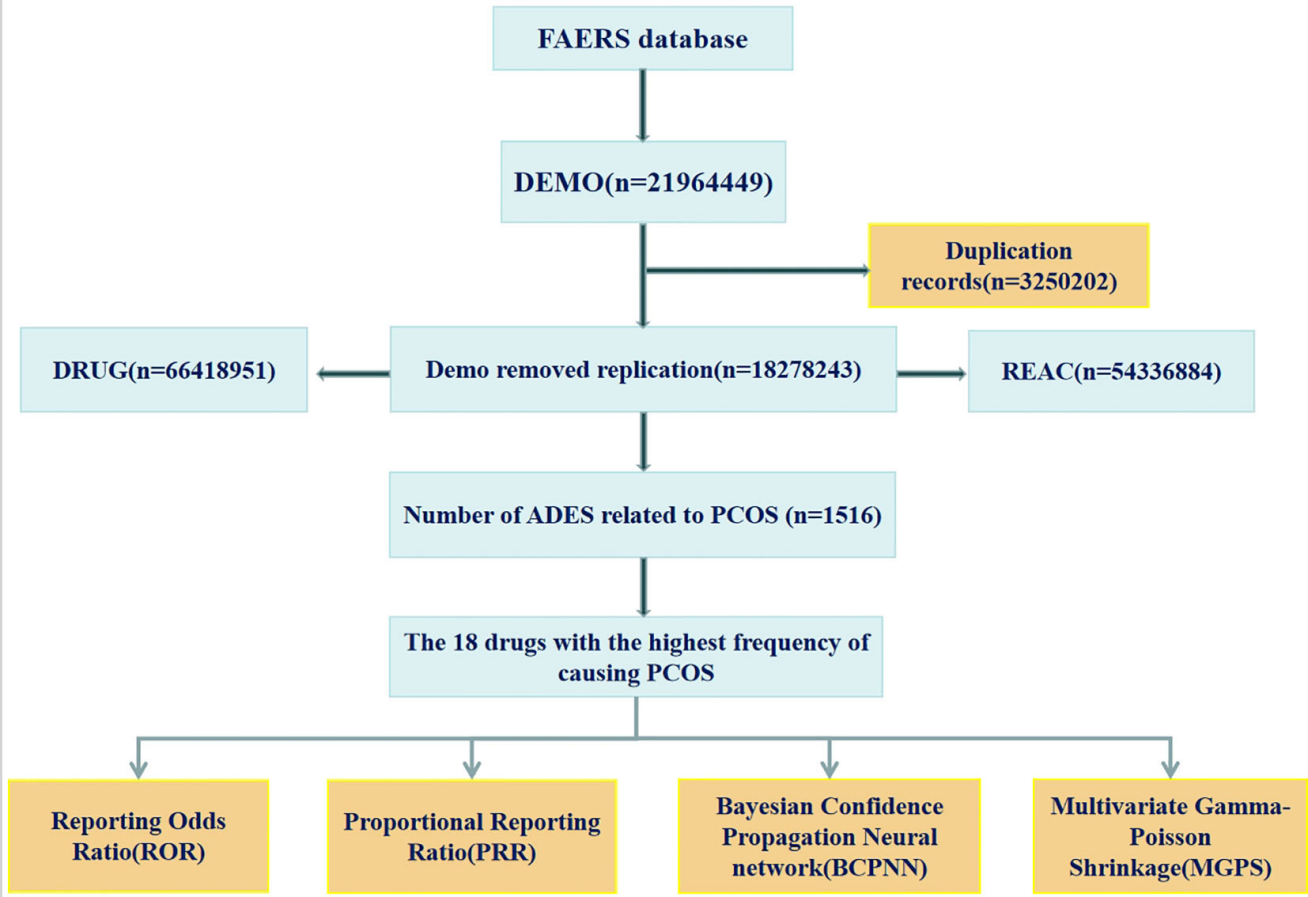
Flowchart of data acquisition and processing for drug-related PCOS.

A total of 1,516 reports of drug-induced polycystic ovary syndrome (PCOS) were retrieved from the FDA Adverse Event Reporting System (FAERS) database spanning Q1–2014 to Q4 2024. These reports include patients’ demographic information such as age, weight, adverse reaction onset time, and adverse reaction outcomes. The data retrieval workflow is illustrated in **Figure 1**.

### 3.2 General information of drug-related polycystic ovary syndrome reports

**Figure 2:**
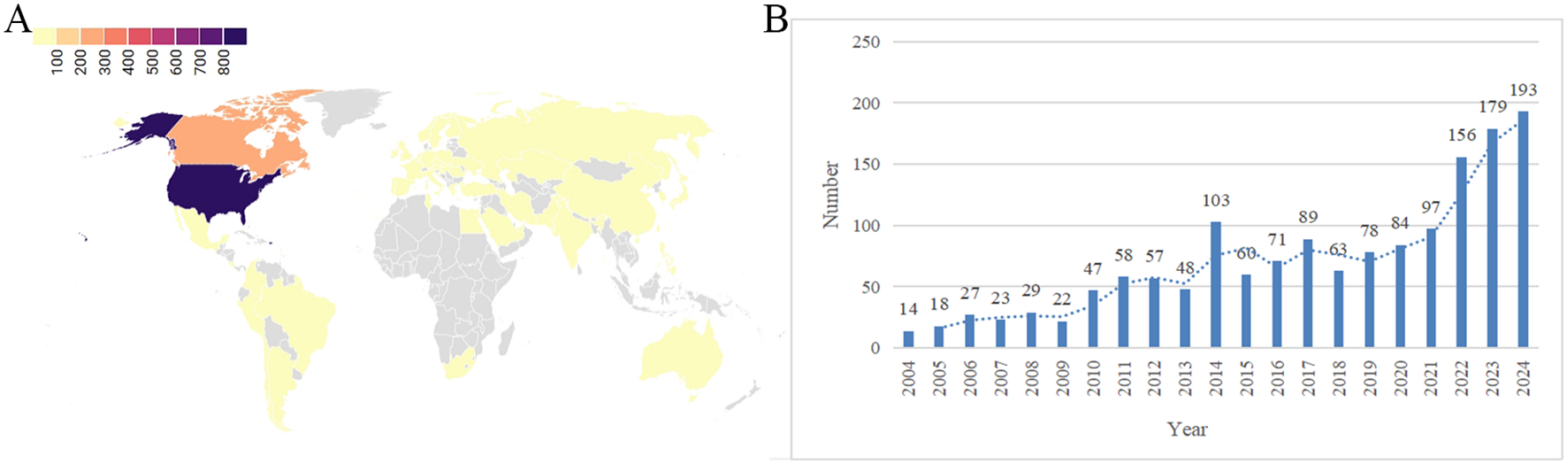
Basic information of drug-related polycystic ovary syndrome reports. **(A)** World Distribution Map of Reporting Countries; **(B)** Number of Reports by Year.

**Figure 3:**
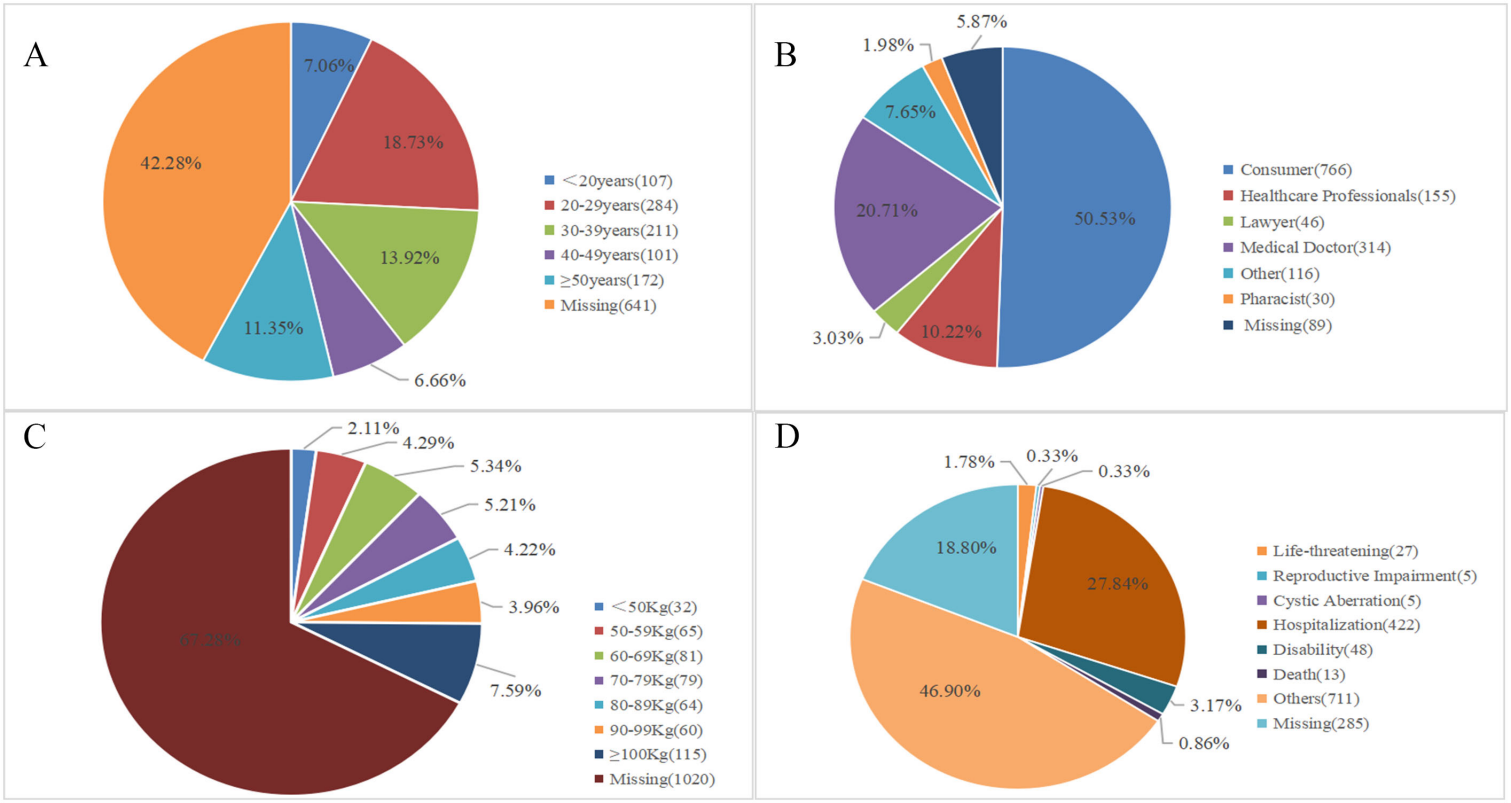
Basic information of drug-related PCOS reports. **(A)** Age Distribution of Drug-related PCOS Reports; **(B)** Distribution of Reporters’ Identities for Drug-related PCOS; **(C)** Weight Distribution in Drug-related PCOS Reports; **(D)** Outcome Distribution of Drug-related PCOS Reports.

The reports of drug-related Polycystic Ovary Syndrome (PCOS) came from 48 countries and regions, with the highest number of reports from the United States, followed by Canada. The number of reports has shown an increasing trend year by year (as shown in **Figure 2**). The majority of the reporters were consumers (50.53%), followed by doctors (20.71%) and healthcare professionals (10.22%). In these reports, the most common age group was 20–29 years (18.73%), followed by 30–39 years (13.92%), and so on. The majority of reported weights were &gt;100kg (7.59%), followed by 60-69kg (5.34%), and others. The outcomes of these reports were primarily categorized as “other” (46.90%), followed by “hospitalization” (27.84%), and so on (as shown in **Figure 3**).

### 3.3 Drug risks and classification for drug-related PCOS

Based on the four methods (ROR, PRR, BCPNN, and MGPS), 18 potential drugs that may induce PCOS were identified as positive. According to the ROR values, the drugs with the highest risk are Mecasermin (ROR = 67.54), followed by Ciclesonide (ROR = 62.1). In terms of frequency ranking, the drugs are: Valproic acid (ROR = 20.78), Olanzapine (ROR = 10.27), Lamotrigine (ROR = 5.25), Quetiapine (ROR = 3.52), Clonazepam (ROR = 12.90), Salbutamol (ROR = 3.18), Montelukast (ROR = 10.29), Levothyroxine (ROR = 3.36), Aciclovir (ROR = 15.96), Budesonide; Formoterol (ROR = 4.26), Tramadol (ROR = 3.88), Aclidinium (ROR = 25.76), Modafinil (ROR = 32.94), Budesonide (ROR = 4.68), Ciclesonide (ROR = 62.1), Ofatumumab (ROR = 3.72), Mecasermin (ROR = 67.54), and Ziprasidone (ROR = 5.10) (as shown in **Table 4**).

**Table 4:** Statistical values and distribution of drugs inducing PCOS.

Drug	Number	ROR (95%CI)	PRR (χ2)	EBGM (EBGM05)	IC (IC025)
Valproic acid	56	20.78 (15.91 - 27.14)	20.75 (1013.71)	20.02 (16.01)	4.32 (3.93)
Olanzapine	39	10.27 (7.47 - 14.11)	10.26 (317.54)	10.02 (7.68)	3.32 (2.86)
Lamotrigine	22	5.25 (3.45 - 8)	5.25 (74.56)	5.19 (3.65)	2.37 (1.77)
Quetiapine	22	3.52 (2.31 - 5.36)	3.52 (39.1)	3.48 (2.45)	1.8 (1.19)
Clonazepam	20	12.9 (8.3 - 20.06)	12.89 (216.5)	12.73 (8.8)	3.67 (3.04)
Salbutamol	19	3.18 (2.02 - 5)	3.18 (28.06)	3.15 (2.16)	1.66 (1.01)
Montelukast	18	10.29 (6.47 - 16.38)	10.28 (149.1)	10.17 (6.9)	3.35 (2.68)
Levothyroxine	14	3.36 (1.99 - 5.69)	3.36 (23)	3.34 (2.15)	1.74 (0.99)
Aciclovir	13	15.96 (9.24 - 27.56)	15.94 (180.49)	15.81 (10.01)	3.98 (3.21)
Budesonide; Formoterol	11	4.26 (2.35 - 7.7)	4.26 (27.2)	4.23 (2.58)	2.08 (1.25)
Tramadol	11	3.88 (2.14 - 7.02)	3.88 (23.34)	3.86 (2.35)	1.95 (1.11)
Aclidinium	9	25.76 (13.37 - 49.64)	25.71 (212.48)	25.56 (14.76)	4.68 (3.76)
Modafinil	9	32.94 (17.09 - 63.49)	32.85 (276.33)	32.66 (18.86)	5.03 (4.11)
Budesonide	8	4.68 (2.34 - 9.37)	4.68 (23.01)	4.66 (2.6)	2.22 (1.25)
Ciclesonide	8	62.1 (30.94 - 124.61)	61.79 (475.96)	61.47 (34.32)	5.94 (4.97)
Ofatumumab	8	3.72 (1.85 - 7.44)	3.71 (15.79)	3.7 (2.07)	1.89 (0.92)
Sodium Chloride	5	8.13 (3.38 - 19.57)	8.13 (31.15)	8.1 (3.89)	3.02 (1.84)
Mecasermin	4	67.54 (25.25 - 180.68)	67.18 (260.1)	67 (29.41)	6.07 (4.77)
Ziprasidone	4	5.1 (1.91 - 13.6)	5.09 (13.13)	5.08 (2.24)	2.35 (1.05)

ROR, Reporting Odds Ratio; PRR, Proportional Reporting Ratio; BCPNN, Bayesian Confidence Propagation Neural Network; MGPS, Multi-item Gamma Poisson Shrinker; EBGM, Empirical Bayesian Geometric Mean; CI, Confidence Interval; χ2, Chi-square; IC, Information Component; IC025, the lower limit of the 95% one-sided confidence interval for IC; EBGM05, the lower limit of the 95% CI for EBGM.

**Figure 4:**
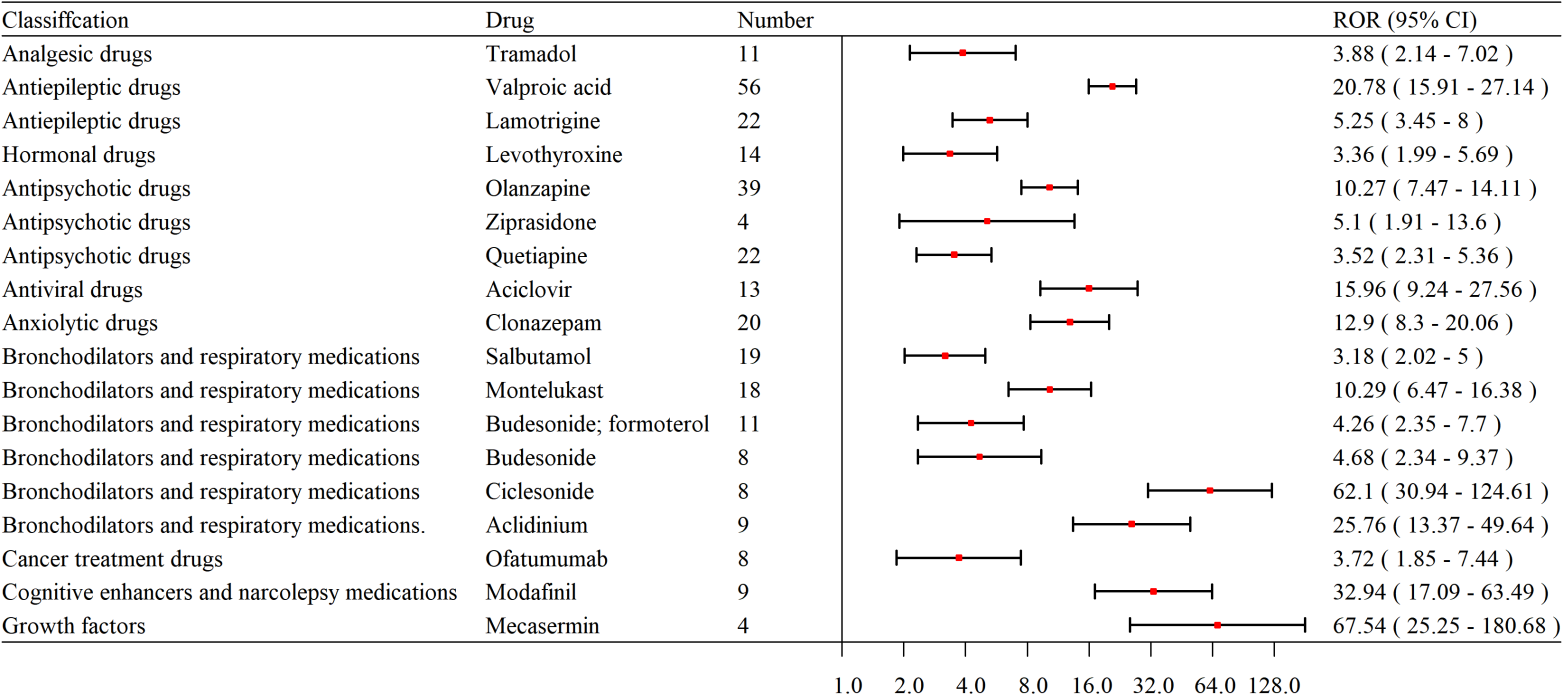
Forest plot of PCOS induced by different drugs under ROR classification. Abbreviation: ROR, Reporting Odds Ratio; CI, Confidence Interval.

The selected high-risk drugs were classified as follows: Bronchodilators and respiratory medications include 6 types, Antipsychotic drugs include 3 types, Antiepileptic drugs include 2 types, Hormonal drugs include 1 type, Analgesic drugs include 1 type, Antiviral drugs include 1 type, Anxiolytic drugs include 1 type, Cancer treatment drugs include 1 type, Cognitive enhancers and narcolepsy medications include 1 type, and Growth factors include 1 type (as shown in **Figure 4**).

### 3.4 Drug-related PCOS induction time

**Figure 5:**
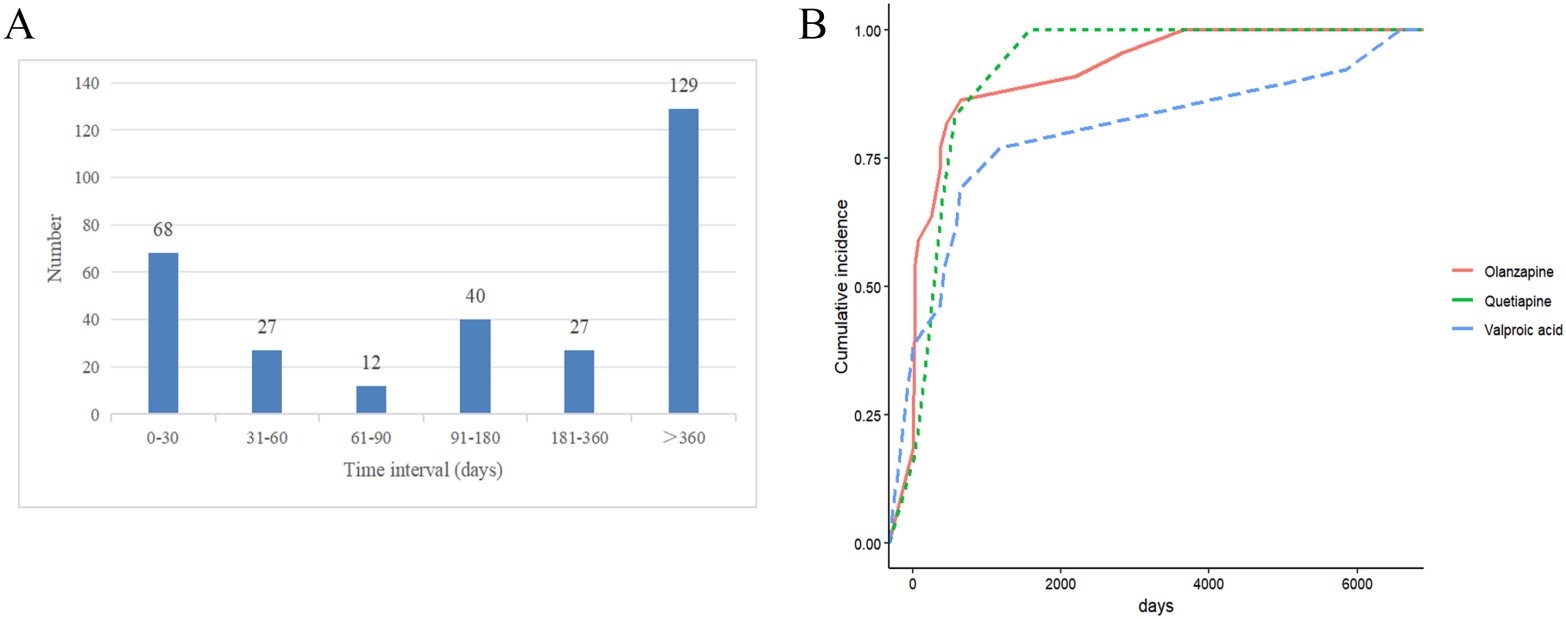
Drug-related PCOS induction time for risk drugs. **(A)** Drug-Induced Onset Time; **(B)** Cumulative Risk Timeline of PCOS Induced by Different Drugs.

Among the reports on drug-related PCOS, 303 reports documented the onset time of drug-induced PCOS. The highest number of cases occurred after &gt;360 days (129 cases), followed by 0–30 days (68 cases) (as shown in **Figure 5**). In this study, we further analyzed the induction time of the top 3 drugs based on signal frequency ranking. For Olanzapine, the median induction time for PCOS was 155.5 days, for Quetiapine, it was 335.00 days, and for Valproic acid, it was 905.00 days (as shown in **Table 5**).

**Table 5:** Time distribution of PCOS induced by different drugs.

Drug	Median (day)	Q1 (day)	Q3 (day)
Olanzapine	155.50	30.00	501.75
Quetiapine	335.00	127.25	826.50
Valproic acid	905.00	465.50	5259.00

Q1,25th percentile; Q3,75th percentile.

## 4 Discussion

PCOS is a common reproductive endocrine disorder that significantly affects women’s reproductive health (18). Its etiology is complex; while genetic and environmental factors are widely regarded as the primary causes, drug-induced PCOS should not be overlooked (19). In this study, we utilized large-scale real-world data from the FAERS database to systematically analyze the potential risk drugs associated with the onset of PCOS. Through screening, we identified 18 drugs that may trigger PCOS, spanning categories such as respiratory drugs, psychiatric medications, antiepileptic drugs, and hormone regulators. Our findings provide important reference information for clinical drug safety evaluations.

Previous studies have shown that long-term use of various psychiatric and antiepileptic drugs may affect female reproductive endocrinology and lead to PCOS (20). In our study, we observed that antiepileptic drugs, such as valproic acid (ROR = 20.78) and lamotrigine (ROR = 5.25), were associated with PCOS. Previous reports have indicated that the risk of PCOS associated with lamotrigine is lower than that associated with valproic acid, which is consistent with our findings (11, 21). Although the exact mechanisms through which these two drugs induce PCOS remain unclear, several small-scale clinical studies have reported PCOS-like symptoms—such as ovulatory dysfunction, hyperandrogenemia, increased body mass index, and insulin resistance—associated with valproic acid and lamotrigine (22–25). Valproic acid may also disrupt the balance of the hypothalamic-pituitary-ovarian (HPO) axis by affecting the secretion of gonadotropin-releasing hormone (GnRH), potentially leading to PCOS or other reproductive endocrine disorders in women (26). Moreover, some studies have suggested that Valproic acid may cause follicular development disorders by disrupting the balance between luteinizing hormone (LH) and follicle-stimulating hormone (FSH), resulting in polycystic ovaries (27). Therefore, female patients using antiepileptic drugs long-term should regularly monitor their endocrine levels and closely observe for any PCOS-related symptoms. In this study, the median onset time for PCOS after using Valproic acid was 905.00 days, which provides an important time reference for clinical management. This study also observed that antipsychotic drugs such as Olanzapine (ROR = 10.27), Ziprasidone (ROR = 5.1), and Quetiapine (ROR = 3.52) were associated with PCOS onset. Previous research has shown that these drugs may trigger metabolic disorders, such as weight gain and insulin resistance, with Olanzapine showing the highest incidence rate (28). Several rat experiments have linked Olanzapine to severe metabolic abnormalities, such as weight gain, insulin resistance, and hyperlipidemia (29–31). In one experiment on female rats, Olanzapine might cause insulin-resistant PCOS by regulating the IGF1/p-AKT/FOXO1 and NF-KB/IL-1β/TNF-α signaling pathways (32). In our study, we also observed the association of the analgesic Tramadol (ROR = 3.88) and the anti-anxiety drug Clonazepam (ROR = 12.9) with PCOS. Tramadol is a centrally acting analgesic that relieves pain by acting on neurotransmitters in the brain and spinal cord and also has some antidepressant effects (33). Clonazepam is a benzodiazepine that enhances the inhibitory neurotransmitter γ-aminobutyric acid (GABA) in the brain to alleviate anxiety and induce sedation (34). However, there is currently very limited research on the relationship between these drugs and PCOS in populations. Modafinil, a drug commonly used to treat narcolepsy, insomnia, and attention deficit disorders, improves alertness and attention by affecting neurotransmitters in the brain (35). Common side effects include headaches, nausea, diarrhea, nervousness, anxiety, indigestion, and insomnia (36). In addition, some studies suggest that modafinil may help maintain wakefulness and enhance alertness by acting on the hypothalamus and regulating the orexin system (37–39). For patients with PCOS, research shows that the hypothalamus may play a significant role in the onset and progression of the condition, with abnormal hormone regulation, such as elevated androgen levels, being closely linked to hypothalamic dysfunction (40). Modafinil’s effects might influence this regulatory process, potentially affecting hormone levels in PCOS patients. However, no studies have yet clearly demonstrated the relationship between modafinil and PCOS, and further research is needed to explore this potential link.

We also observed that bronchodilators and respiratory medications such as Salbutamol (ROR = 3.18), Montelukast (ROR = 10.29), Budesonide; formoterol (ROR = 4.26), Budesonide (ROR = 4.68), Ciclesonide (ROR = 62.1), and Aclidinium (ROR = 25.76) were associated with PCOS. Budesonide and Ciclesonide are steroid medications (41)that may interfere with hormonal balance in the body. Long-term use may lead to weight gain, insulin resistance, and other issues that are associated with the onset and exacerbation of PCOS (42, 43). Salbutamol, a β2-adrenergic receptor agonist, is primarily used to relieve bronchoconstriction (44). Previous studies have shown that, during hypoglycemic reactions, salbutamol may affect hormone secretion and the counterregulatory response by acting on the hypothalamus, specifically by regulating lactate levels and the hormonal response to hypoglycemia (45). Since the occurrence of PCOS is related to hypothalamic regulation of hormones, this suggests a possible link between salbutamol and PCOS. However, no studies have yet confirmed this hypothesis. Montelukast is a leukotriene receptor antagonist widely used in the treatment of allergic diseases and asthma. Leukotrienes play a crucial role in immune responses, particularly in allergic inflammation (46). Studies suggest that abnormal activation of leukotrienes may contribute to the chronic low-grade inflammation observed in PCOS patients (47). This mechanism may indirectly relate to the development of PCOS, but further research is needed to confirm this. Aclidinium is an anticholinergic drug that works by inhibiting the parasympathetic nervous system, reducing airway constriction and secretion production. It is commonly used in the treatment of chronic obstructive pulmonary disease (COPD) (48). Additionally, Aclidinium has been shown to possess anti-inflammatory properties (49). PCOS is closely associated with chronic low-grade inflammation; however, the direct impact of Aclidinium on PCOS still requires further research for validation. Currently, there is no research directly linking the use of salbutamol, montelukast, and aclidinium with the occurrence of PCOS. Therefore, future clinical trials will help further explore the relationship between them.

We also observed a potential association between bronchodilators and respiratory system medications, such as salbutamol (ROR = 3.18), montelukast (ROR = 10.29), budesonide (ROR = 4.68), formoterol (ROR = 4.26), ciclesonide (ROR = 62.1), and aclidinium (ROR = 25.76), and PCOS (polycystic ovary syndrome). Budesonide and ciclesonide are corticosteroids (40) that may interfere with the body’s hormonal balance. Long-term use may lead to weight gain, insulin resistance, and other issues related to the onset and exacerbation of PCOS (41, 42) (43). Previous studies suggest that salbutamol may influence hormone secretion and counter-regulatory responses during hypoglycemic reactions by acting on the hypothalamus, particularly by regulating lactate levels and hormone reactions (44). Montelukast, a leukotriene receptor antagonist, is widely used to treat allergic diseases and asthma. Leukotrienes play an important role in immune responses, particularly in allergic inflammation (45). Studies indicate that abnormal activation of leukotrienes may contribute to the chronic low-grade inflammation seen in PCOS patients (46). This mechanism may indirectly relate to the development of PCOS, but further research is needed to confirm this. Aclidinium, an anticholinergic drug, is commonly used to treat chronic obstructive pulmonary disease (COPD) (47) by inhibiting the parasympathetic nervous system to reduce airway constriction and secretion. Additionally, aclidinium has anti-inflammatory properties (48). Given that PCOS is closely related to chronic low-grade inflammation, further studies are needed to determine whether aclidinium has a direct impact on PCOS. Currently, no studies have directly linked the use of salbutamol, montelukast, and aclidinium to the occurrence of PCOS. Therefore, future clinical trials will help further explore the relationship between these medications and PCOS.

Aciclovir is an antiviral drug commonly used to treat infections caused by the herpes simplex virus, such as shingles and mouth ulcers (50). Aciclovir works by converting into its active form, aciclovir triphosphate, which competitively inhibits viral DNA polymerase, preventing viral replication, and has minimal impact on human cells with low toxicity (51). While aciclovir is highly effective against viruses, its effects on the immune system and potential relationship with endocrine disorders have not been thoroughly studied. Furthermore, there is no clear research establishing a direct relationship between aciclovir and PCOS. Ofatumumab is a monoclonal antibody used to treat certain types of blood cancers, such as chronic lymphocytic leukemia, by targeting B cells, inducing cell death, and modulating immune responses (52). Ofatumumab is also used to treat certain autoimmune diseases (53). There is currently no research confirming a direct link between Ofatumumab and PCOS. However, since PCOS has been associated with chronic low-grade ovarian inflammation (54), this mechanism may relate to the action of Ofatumumab, although further experimental studies are required to validate this hypothesis. Mecasermin is a recombinant human insulin-like growth factor 1 (IGF-1) that promotes skeletal and soft tissue growth and enhances cell proliferation, metabolism, and development by stimulating growth factor receptors (55). Previous studies have shown that elevated IGF-1 activity may lead to chronic anovulation, insulin resistance, and increased adrenal androgen secretion, causing PCOS-like symptoms (56). Another study indicated that chronic inflammation of tissue cells caused by IGF-1 could be one of the mechanisms of chronic inflammation in PCOS patients (57). Furthermore, another study found that miR-323-3p targeting IGF-1 regulates steroidogenesis and CCs activity, potentially improving CCs dysfunction in PCOS (58). Therefore, Mecasermin, as an IGF-1 class drug, may be associated with the onset of PCOS, and further studies could explore its specific mechanisms of action.

Although this study provides us with an analysis of the correlation between drug use and the occurrence of PCOS, it also faces potential influences from indication bias and confounding factors caused by comorbidities or concomitant medication use. Firstly, we must point out that there may be indication bias, which could influence our results. For example, valproate sodium, a commonly used medication for treating bipolar disorder, is often prescribed to patient populations with mental illnesses in clinical practice. However, studies have shown that bipolar disorder itself may be associated with endocrine disorders, which are considered major risk factors for PCOS (59, 60). Therefore, the use of valproate sodium is not just a result of treating bipolar disorder itself, but more a reflection of the endocrine disease state in these patients, which may directly or indirectly increase the risk of PCOS. Additionally, another potential source of bias could arise from confounding factors associated with comorbidities or the use of multiple medications. For instance, many patients with depression are on long-term antipsychotic medications, and a significant proportion of them also suffer from obesity, which is a major risk factor for PCOS. At the same time, some patients may be receiving treatments with antidiabetic drugs, antihypertensive medications, or hormonal therapies. These medications may interact with each other, influencing their pharmacological effects and potentially altering the incidence. Moreover, the FAERS database relies on voluntary reports, which may lead to underreporting, especially of events deemed insignificant or mild by healthcare professionals or patients. As a result, FAERS cannot provide precise incidence rates nor establish causal relationships between drugs and diseases. Furthermore, while proportional imbalance analysis reveals statistical associations, it does not account for confounding factors or the temporal sequence of events. The lack of detailed patient-level information, such as comorbidities, concomitant treatments, or pre-existing metabolic conditions, further complicates causal inferences. Based on the above analysis, future research should focus on overcoming the challenges posed by reporting bias and confounding factors, particularly by integrating prospective studies, clinical data, and data from a broader patient population to further validate the relationship between drugs and PCOS. By combining multiple data sources, researchers will be better equipped to clarify the causal relationship between drug treatments and the occurrence of PCOS, providing more comprehensive and precise guidance for clinical practice.

## 5 Conclusion

With its vast data volume, wide coverage, and open access, the FAERS database has become an important resource for studying adverse drug reactions. Through the FAERS database, we identified 18 drugs that may contribute to the occurrence of PCOS. These drugs are primarily related to the nervous system, followed by respiratory system medications and other types of drugs. This study provides a practical perspective for developing drug safety strategies and addressing drug-related harm. Future research should combine real-world data with experimental validation to develop a comprehensive safety profile for potential drugs related to PCOS, thereby supporting safer treatment options.

## Data Availability

The original contributions presented in the study are included in the article/supplementary material. Further inquiries can be directed to the corresponding author.
